# High-dose extended-field radiotherapy plus chemotherapy improved survival in extranodal NK/T-cell lymphoma in a real-life setting: results from the multicenter *T-Cell Brazil Project*

**DOI:** 10.1038/s41598-022-25034-3

**Published:** 2022-11-29

**Authors:** Luís Alberto de Pádua Covas Lage, Pedro Paulo Faust Machado, Cadiele Oliana Reichert, Eliana Miranda, Hebert Fabrício Culler, Sheila Aparecida Coelho da Siqueira, Renata de Oliveira Costa, Dênis Ricardo Miyashiro, José Antônio Sanches, Vanderson Rocha, Carlos Sérgio Chiattone, Juliana Pereira

**Affiliations:** 1 Department of Hematology, Hemotherapy and Cell Therapy, Faculty of Medicine - University of São Paulo (FM-USP), São Paulo, SP Brazil; 2 Laboratory of Medical Investigation in Pathogenesis and Directed Therapy in Onco-Immuno-Hematology (LIM-31), Hospital das Clínicas da Universidade de São Paulo (HC-FMUSP), Avenue Dr. Enéas de Carvalho Aguiar, 155 – Ambulatory Building - 1st. Floor, Room 61, Cerqueira César, São Paulo, SP 05403-000 Brazil; 3 Laboratory of Medical Investigation in Immunology and Histocompatibility (LIM-19), University of São Paulo (USP), São Paulo, SP Brazil; 4 Department of Hematology and Hemotherapy, University of Campinas (Unicamp), Campinas, São Paulo, SP Brazil; 5 Department of Pathology, University of São Paulo (USP), São Paulo, SP Brazil; 6 Department of Hematology and Hemotherapy, Faculdade de Ciências Médicas de Santos (FCMS), Centro Universitário Lusíada (Unilus), Santos, São Paulo, SP Brazil; 7 Department of Dermatology, Faculty of Medicine – University of São Paulo (FM-USP), São Paulo, SP Brazil; 8 Fundação Pró-Sangue, Blood Bank of São Paulo, São Paulo, SP Brazil; 9 Churchill Hospital, Oxford University, Oxford, UK; 10 Department of Hematology and Hemotherapy, Faculdade de Ciências Médicas da Santa Casa de São Paulo (FCMSC-SP), São Paulo, SP Brazil; 11 Hospital Samaritano, São Paulo, SP Brazil; 12 Hospital Alemão Oswaldo Cruz (HAOC), São Paulo, SP Brazil

**Keywords:** Cancer therapy, Haematological cancer, Haematological cancer, Risk factors

## Abstract

Extranodal natural-killer/T-cell lymphoma (ENKTL) is a rare and aggressive Epstein-Barr virus related mature T-cell and natural-killer malignancy. Although highly prevalent in South America, few studies covering data from this geographic location have been published. Therefore, this study aims to report clinical characteristics, prognostic factors, and outcomes in a multicenter cohort of ENKTL patients from Brazil. This retrospective, observational and multicenter study included 98 ENKTL patients treated during two decades in Brazil. Data were extracted from the *T-Cell Brazil Project* database. In our cohort, 59/98 patients (60.2%) were male, with a median age of 50 years. Sixty-two patients (63.3%) had B-symptoms, 26/98 (26.5%) had *Eastern Cooperative Oncology Group* scale ≥ 2; 16/98 (16.3%) presented extranasal disease and 34.7% (34/98) were advanced-stage (Ann Arbor/Cotswolds III/IV). The median follow-up for the whole cohort was 49 months, with an estimated 2-year overall survival (OS) and progression-free survival (PFS) of 51.1% and 17.7%, respectively. In early-stage disease (IE/IIE), the median OS was 21.8 months for patients treated with concurrent radiotherapy plus chemotherapy (CCRT-VIPD [etoposide/vp-16, ifosfamide, cisplatin and dexamethasone), 16.2 months for sequential chemoradiotherapy (SCRT) followed by asparaginase-based regimens, and 56.7 months for SCRT followed by CHOP-like (cyclophosphamide, doxorrubicin, vincristine and prednisone) treatments, *p *= 0.211. CCRT was associated with higher rates of early-mortality, hematological toxicity, and mucositis. Median OS was 8.2 months for patients with advanced-stage disease receiving regimens containing asparaginase compared to 3.2 months for anthracycline-based therapy, *p *= 0.851. Chemo-radiotherapy (CRT) regimens demonstrated better OS (*p *= 0.001) and PFS (*p *= 0.007) than chemotherapy alone. Multivariate analysis revealed anemia, relapsed/refractory (R/R) disease and radiotherapy omission as poor outcome predictors for OS. Lymphopenia and radiotherapy omission adversely affected PFS. Concerning progression of disease within 24-months (POD-24), clinical stage III/IV was a poor outcome predictor. In this real-life Brazilian cohort, ENKTL presented dismal outcomes. Radiation therapy was an independent factor for increased OS and PFS, but CCRT regimens were associated with higher toxicities. Polychemotherapy based on anti-multi drug resistant agents was not associated with survival benefit in either early or advanced-stage disease in our patient cohort.

## Introduction

Extranodal natural killer-/T-cell lymphoma (ENKTL) is a mature T-cell or natural killer (NK)-cell lymphoma recognized as a single entity in the 2016-World Health Organization (WHO) Classification for Hematopoietic and Lymphoid Tissue Neoplasms^1^. Formely described “*nasal-type*”; the upcoming 5th new WHO edition dropped this qualifier from its name in WHO-HAEM5^2^. Commonly arising in the midfacial line and compromising the upper aero-digestive structures, about one-third of cases may present extranasal origin affecting the skin, soft tissues, testes, gastrointestinal tract, and lungs^1,3^. ENKTL accounts for 10% of all mature T-cell lymphomas^4^, being more prevalent in East Asia and South America^1,3^. It is an angiocentric malignancy, universally associated with the Epstein-Barr virus (EBV) infection and has an aggressive clinical behavior with dismal prognosis^1^**.**

ENKTL usually affects patients in a median age of 50 years, with a male predominance^1,3^. Clinically, it presents as ulcerative, destructive, and exuberant necrotic lesions. Tissue necrosis results in delayed diagnosis, making multiple biopsies often necessary. Secondary naso-sinusal infection and bone erosion leading to perforation of the hard palate and the nasal septum are frequently found^5^**.** Historically, this disorder was recognized as “*lethal granuloma of the midline*”, and the main differential diagnosis comprises chronic granulomatous diseases affecting the face, such as cutaneous-mucosal leishmaniasis, tuberculosis, leprosy, South-American blastomycosis, mucormycosis and Wegener’s granulomatosis^6^. At diagnosis, while most cases have early-stage disease (IE/IIE), disseminated involvement of multiple extranodal sites may occur in up to 25% of patients. Hemophagocytic syndrome (HPS) is a well-recognized complication and central nervous system (CNS) dissemination can also occur. Although rare, progression to aggressive NK-cell leukemia has been described^1,7^.

About 90% of ENKTL derives from NK-cells, with 10%-15% presenting with a T-CD8 + cytotoxic phenotype^1,5,8^. Most cases express cytoplasmic CD3 (ε-chain) and cytotoxic granules, such as TIA-1, perforin, and granzyme-B, and CD56 (NCAM – *neural-cell adhesion molecule*). The marker CD30 (Ki-1) may be found in approximately 30–40% of cases, and EBV positivity on neoplastic cells is universally present^1,3^. Genomic-molecular recurrent abnormalities, such as chr6q21-23 deletions, *STAT3* activation and *JAK/STAT* pathway deregulation are present in 50% of cases^9–11^. Other mutations involving the tumor suppressor’s genes *BCOR*, *DDX3X* and *TP53* also contribute for the ENKTL oncogenesis^12–14^.

The tumor cells of the ENKTL present high P-glycoprotein (Pgp) concentration, which confers a multidrug resistant (MDR) phenotype and resistance to anthracycline-based regimens, the most used treatment in clinical practice for mature T-cell lymphomas^4,15,16^. Chemotherapy agents attempting to overcome MDR mechanisms such as platinum analogues, ifosfamide, etoposide and asparaginase have demonstrated better responses and improved outcomes when compared to anthracyclines^17–21^. Since ENKTL is highly sensitive to radiation, high-dose extended-field radiotherapy (EF-RT) with 50–60 Gy has become a backbone for treatment of early-stage disease^17,22,23^.

Although prevalent in South America, data from ENKTL cohorts are scarce in Brazil. Hence, in this study, we aimed to describe clinical characteristics, outcomes, prognostic factors, response to therapy and treatment related toxicities in a multicenter cohort of patients with ENKTL from Brazil.

## Patients and methods

### Study design, ethical issues and casuistic

This is a retrospective, observational and multicenter study, incorporating data from 98 ENKTL patients registered in 20 Brazilian centers from the *T-Cell Brazil Project*, including data from January 2000 to December 2021. This study was approved by all the involved center’s Local Ethics Committees with waiver of the Informed Consent Form.

Eletronic medical records were reviewed retrospectively and clinical-epidemiological, laboratory and histopathological data were uploaded into RedCap plataform (https://lnh-celulast.hemocentro.unicamp.br) by each center and managed centrally by the scientific commitee. The data were transferred into a Microsoft Excel workbook for subsequent statistical analysis.

### Histopathology and immunohistochemistry

All patients had a biopsy-proven diagnosis of ENKTL based on Hematoxylin-Eosine (HE) staining, immunohistochemistry (IHC) and EBV testing on tumor tissues by in situ* hydridization* (ISH) or by IHC for LMP-1 protein expression performed according to local practice. An initial analysis of HE slides showed atypical lymphoid tissue infiltration by small to medium-sized cells, variable degree of mitosis and necrosis, and exuberant angioinvasion and angiodestruction. IHC study was performed with the monoclonal antibodies CD45 (Dako, 2B11 + PD7/26, 1/2000), Ki67 (Dako, J55, 1/1600), CD2 (Monossan, AB75M, 1/200), CD3 (Dako, F7.2.38, 1/500), CD4 (Spring, SP5, 1/400), CD7 (Novocastra, CD7-272, 1/3000), CD8 (Dako, C8/144B, 1/800), Ki-1/CD30 (Cell Marque, Ber-H2, 1/1000), CD56 (Sigma-Aldrich, RM315, 1/1000), TIA-1 (Abcam, EPR9304, 1/200), Granzyme-B (Abcam, BLR022E, 1/500), Perforin (ThermoFisher, MA5-12,469, 1/500) and ISH for EBV (*ZytoFast Plus CISH*© kit—ZytoVision, Bremerhaven, Germany) to confirm ENKTL diagnosis.

### Database and staging

Baseline variables accessed at diagnosis included age, gender, site of lymphoma (nasal-type versus extranasal), performance status by *Eastern Cooperative Oncology Group* (ECOG), lactic dehydrogenase (LDH), β2-microglobulin (β2-MG), hemoglobin (Hb), blood counts, albumin, globulin, HIV, HTLV-1, hepatitis B and C serology, EBV viral load in peripheral blood, images, bulky ≥ 7 cm, B-symptoms, bone marrow and central nervous system infiltration, clinical stage, prognostic score systems (PINK and PINK-E), tumor necrosis, CD30 expression, secondary naso-sinusal infection, up-front chemotherapy regimen, radiotherapy and hematopoietic stem cell transplantation (HSCT).

Lymphoma staging was performed according to institutional imaging protocols following the 2014-Lugano Recommendations^24^. Computarized tomography (CT) scans and/or CT with positron emission with 18-fluorodeoxyglucose (18-FDG-PET CT) with unilateral bone marrow biopsy were perfomed. Magnetic resonance imaging (MRI) of face and brain were performed at discretion of the physician. Selected patients underwent lumbar puncture in the presence of neurological symptoms.

### Treatment modalities, response assessment and follow-up

Patients were treated according to local protocols, using concurrent chemotherapy and radiotherapy (CCRT-VIPD)^25^, sequential chemoradiotherapy (SCRT) with high-dose extended-field radiotherapy (EF-RT) followed by anthracycline-based regimens (CHOP-like), or with chemotherapy schemes containing asparaginase, such as SMILE^26^, P-GEMOX^27^, GELOX^20^ or AspaMethDex^28^. Few cases were managed with anti-MDR drug-based regimens not containing asparaginase, such as the protocols GEMOX/GEMOD^29^, GDP^30^ and DeVIC^31^.

Central nervous system prophylaxis was indicated according to local practice, but formally recommended for paranasal sinuses, orbit, Waldeyer's ring, testes, and skull base involvement. These cases underwent four intrathecal injections with 12 mg methotrexate and 2 mg dexamethasone. Response assessment was based on 2014-Lugano Response Criteria for non-Hodgkin’s Lymphomas^24^. The follow-up was carried out according to local practice.

### End points and statistical analysis

The overall response rate (ORR), including complete response (CR), partial response (PR), overall survival (OS), progression-free survival (PFS) and disease progression within 24 months from the beginning of therapy (POD-24) were calculated for the whole cohort and for the main treatment groups.

Descriptive analysis was carried out according to the variables evaluated; categorical variables were presented in absolute (N) and relative values (%). Continuous variables were showed as measures of central tendency (median), dispersion (IqR) and position. For laboratory variables, cut-off included values above or below normal ranges.

The cohort follow-up time was calculated using the reverse Kaplan–Meier method. OS, PFS and POD-24 curves were elaborated using the Kaplan–Meier method and the Log-Rank test was used to assess the relationship between the variables and survival outcomes. OS was calculated from the date of diagnosis (considered the first lymphoma positive biopsy date for) to death, PFS from the date of diagnosis until disease progression, death, or last follow-up. POD-24 was considered as the percentage of cases presenting progression of disease within 24 months of starting up-front treatment.

Data were censored at the last follow-up. Univariate analysis was performed by Cox semi-parametric test and multivariate analysis by Cox regression or proportional ratio model. Variables with *p-*value ≤ 0.10 at univariate analysis were included in the final model. The results were showed in hazard ratio (HR) and 95% Confidence Interval (95% CI). All analyses were performed using *SPSS statistical software*® (IBM SPSS version 22.0) for Windows and a *p-*value ≤ 0.05 was considered statistically significant.

### Ethics approval and consent to participate

The local Ethics Committees of all participanting institutions (Research Ethics Committee of São Paulo University, Research Ethics Committee of Campinas State University and Research Ethics Committee of Federal University of São Paulo) approved this study in 2015. All procedures were in accordance with the ethical standards of the comittees responsibles for human experimentation (institutional and national) and with the Helsinki Declaration of 1975, as revised in 2008. The Ethics Comittees for Local Research (São Paulo University, Campinas State University and Federal University of São Paulo) waived the application of Informed Consent Forms (ICF).

## Results

### Baseline patient’s characteristics

Ninety-eight ENKTL patients were included in the final analysis. The median age was 50 years (IqR: 40–58 years), 20.4% (20/98) were elderly (≥ 60 years) and 60.2% (59/98) were male. Sixty-eight percent (66/98) were Hispanic, 25.5% (25/98) Asian, and 6.5% (6/98) African American. Sixteen patients (16.3%) had extra-nasal origin and 34/98 (34.7%) presented advanced clinical stage (Ann Arbor/Cotswolds III/IV), 2/98 (2%) had bone marrow infiltration and central nervous system (CNS) involvement by lymphoma. The CNS involvement was diagnosed by brain magnetic resonance imaging (MRI) and cerebrospinal fluid (CSF) analysis, including oncotic cytology and immunophenotyping by flow cytometry. Most patients, making up 63.3% (62/98) had B-symptoms, 26/98 (26.5%) presented ECOG ≥ 2 and 7/98 (7.1%) showed bulky disease ≥ 7 cm. Infection in the tumor site was seen in 41.8% (41/98), high-risk PINK score was observed in 29/98 cases (30.2%), and high-risk PINK-E score occurred in 6/65 cases (9.2%). Hemophagocytic syndrome according to HLH-2004 Consensus was refered in 2/98 (2%) patients^32^. The baseline laboratory and histopathological characteristics of all patients are summarized in Table 1.

**Table 1:** Laboratory and histopathological characteristics for 98 ENKTL Brazilian patients.

Variable	Median (IqR)	Categorization – N (%)
Hemoglobin (g/dL)	12.3 (10.4–13.8)	Hb < 12 g/dL – 42/98 (42.8%)
Leukocytes (× 10^9^/L)	6.05 (4.06–8.82)	Leukopenia – 23/98 (23.4%)
		Leukocytosis – 16/98 (16.3%)
Neutrophils (× 10^9^/L)	3.80 (2.40–6.60)	Neutropenia – 04/98 (4.0%)
		Neutrophilia – 16/98 (16.3%)
Lymphocytes (× 10^9^/L)	1.10 (0.71–1.60)	Lymphopenia – 37/98 (21.4%)
		Lymphocytosis – 02/98 (2.0%)
Monocytes (× 10^9^/L)	0.50 (0.30–0.80)	Monocytopenia – 11/98 (11.2%)
		Monocytosis – 13/98 (13.2%)
N/L ratio	3.5 (2.3–5.8)	–
L/M ratio	2.1 (1.5–3.3)	–
Platelets (× 10^9^/L)	233 (176–304)	Thrombocytopenia – 34/98 (34.6%)
		Thrombocytosis – 06/98 (6.1%)
LDH (U/L)	341 (219–462)	LDH ≥ UVN – 21/91 (23.1%)
Β2-microglobulina (mg/dL)	2.80 (1.70–3.90)	Β2mg ≥ UVN – 39/51 (76.5%)
Albumin (g/dL)	3.50 (2.90–4.10)	Hipoalbuminemia – 35/77 (45.5%)
Globulin (g/dL)	1.80 (1.50–2.40)	Hipogamaglobulinemia – 18/37 (48.6%)
Qualitative EBV PCR in PB	–	Positive – 41/65 (63.1%)
Quantitative EBV PCR in PB	Log 2.7 (2.4–2.9)	–
Extensive necrosis (≥ 50%)	–	40/98 (40.8%)
CD30 positivity	–	35/93 (37.6%)
CD56 positivity	–	95/98 (96.9%)

*IqR* Interquartile interval, *Hb* Hemoglobin, *N/L* Neutrophils/lymphocytes ratio, *L/M* Lymphocytes/monocytes ratio, *LDH* Lactic dehydrogenase, *β2mg* Beta2-microglobulin, *EBV* Epstein-Barr virus, *PCR* Polymerase chain reaction, *PB* Peripheral blood, CD Cluster designation.

### Up-front treatment modalities

Ninety-three (94.9%) of 98 ENKTL patients were submitted to at least one line of treatment, and 5/98 (5.1%) died during the staging procedures without receiving any therapy. Fifty-three (54.1%) patients experienced chemoradiotherapy (CRT) in concurrent (CCRT) or in sequential (SCRT) modalities, 37/98 (37.8%) received only chemotherapy and 3/98 (3.1%) underwent isolated EF-RT.

Ninety patients were submitted to chemotherapy, however, in one case, information regarding the chemotherapy protocol used was unobtainable. Therefore, among the 89 patients with known chemotherapy protocols, 32 cases (36%) were treated with SCRT based on anthracyclines (CHOP-like), 31/89 (34.8%) with asparaginase-based regimens, 21/89 (23.6%) with CCRT-VIPD, and only 5/89 (5.6%) received miscellaneous protocols based on anti-MDR drugs without asparaginase. Fifty-six (57.1%) patients with early-stage disease (IE/IE) received EF-RT at median dose of 54 Gy (IqR: 50–60 Gy) with curative purpose, 5/98 (5%) were submitted to ASCT, being 3/5 (60%) in up-front therapy and 2/5 (40%) in second line for relapsed or refractory (R/R) disease. Among the two ENKTL patients diagnosed with secondary hemophagocytic lymphohistiocytosis (HLH) one received pulse therapy with methylprednisolone 1 g/day I.V. for 3 days, followed by P-GEMOX regimen, and the other case was managed exclusively with specific therapy for ENKTL with SMILE polychemotherapy.

### Outcomes

The overall response rate (ORR) was 51.0% (95% CI: 41.1–60.9) and 19/98 (19.3%) of cases (95% CI: 11.5–27.2) were primary chemorefractory. Refractory/Relapsed (R/R) disease was observed in 40.8% [40/98] (95% CI: 31.4–50.7). Thirty-two cases (80%) presented refractoriness/early relapsed disease (≤ 12 months from remission) and only 8/40 (20%) presented relapses for more than 12 months after achieving remission (late-relapses). POD-24 rate was 35.7% (95% CI: 26.7–45.5) and the median time to relapse/progression was 4.9 months. Table 2 summarizes the main clinical, laboratory and therapeutic characteristics of ENKTL patients categorized by the pattern of response to up-front therapy (responsive, refractory, or relapsed disease). Primary refractory patients had a higher percentage of high-risk PINK score (*p *= 0.035), higher rates of secondary nasosinusal infection (*p *< 0.001), higher frequency of lymphopenia (*p *= 0.085) and higher rates of EF-RT omission in the up-front therapeutic regimen (*p *= 0.002). However, there was no difference regarding the percentage of cases treated with anthracycline-based chemotherapy (*p *= 0.963).

**Table 2:** Clinical-laboratory characteristics and therapeutic modalities in 93 ENKTL patients categorized by response to up-front treatment.

Characteristic	Non-R/R (N = 53)	Refractory (N = 19)	Relapsed (N = 21)	*p-*value
Age ≥ 60 years	13 (24.5%)	3 (15.8%)	3 (14.3%)	0.516
Extranasal disease	6 (11.3%)	4 (21.1%)	3 (14.3%)	0.597
CS III/IV	15 (28.3%)	9 (47.4%)	6 (28.6%)	0.303
ECOG ≥ 2	16 (30.2%)	5 (26.3%)	3 (14.3%)	0.369
PINK high-risk	13 (24.5%)	10 (52.6%)	4 (19.0%)	0.035
B-Symptoms	35 (66.0%)	13 (68.4%)	11 (52.4%)	0.480
Bulky ≥ 7 cm	1 (1.8%)	2 (10.5%)	3 (14.3%)	0.106
Nasal infection	21 (39.6%)	17(89.4%)	11 (52.3%)	< 0.001
Hb < 120 g/L	23 (43.4%)	9 (47.3%)	9 (42.8%)	0.948
Leukopenia	11 (20.7%)	6 (31.6%)	5 (23.8%)	0.635
Neutropenia	2 (3.8%)	1 (5.2%)	1 (4.8%)	0.956
Lymphopenia	20 (37.8%)	10 (52.6%)	4 (19.0%)	0.085
Thrombocytopenia	7 (13.2%)	3 (14.2%)	1 (4.8%)	0.499
Albumin < 3.5 g/dL	17/44 (38.6%)	9/15 (60.0%)	5/14 (35.7%)	0.299
EBV-DNA positivity	26/39 (66.7%)	8/14 (57.1%)	10/12 (83.3%)	0.354
No EF-RT	21 (39.6%)	13 (68.4%)	3 (14.3%)	0.002
**Chemo-regimen**				
CCRT/VIPD	11/48 (22.9%)	5/16 (31.3%)	5/20 (25.0%)	0.963
Asparaginase	18/48 (37.5%)	6/16 (37.5%)	7/20 (35.0%)	
CHOP-like	19/48 (39.6%)	5/16 (31.3%)	8/20 (40.0%)	

*R/R* Relapsed/refractory disease, *CS* Clinical stage (Ann Arbor/Cotswolds), *ECOG* Eastern Cooperative Oncology Group scale, *PINK* Prognostic index for nasal NK lymphoma, *LDH* Lactic dehydrogenase, *Hb* Hemoglobin, Leukopenia < 4.0 × 10^9^/L, Neutropenia < 1.5 × 10^9^/L, Lymphopenia < 0.9 × 10^9^/L, Thrombocytopenia < 150 × 10^9^/L, *EBV DNA* Epstein-Barr virus DNA by qualitative PCR in peripheral blood sample, *EF-RT* Extended-field radiotherapy, *CCRT/VIPD* Concurrent chemoradiotherapy/etoposide, ifosfamide, cisplatin and dexamethasone, *CHOP* Cyclophosphamide, doxorubicin, vincristine and prednisone.

**Figure 1:**
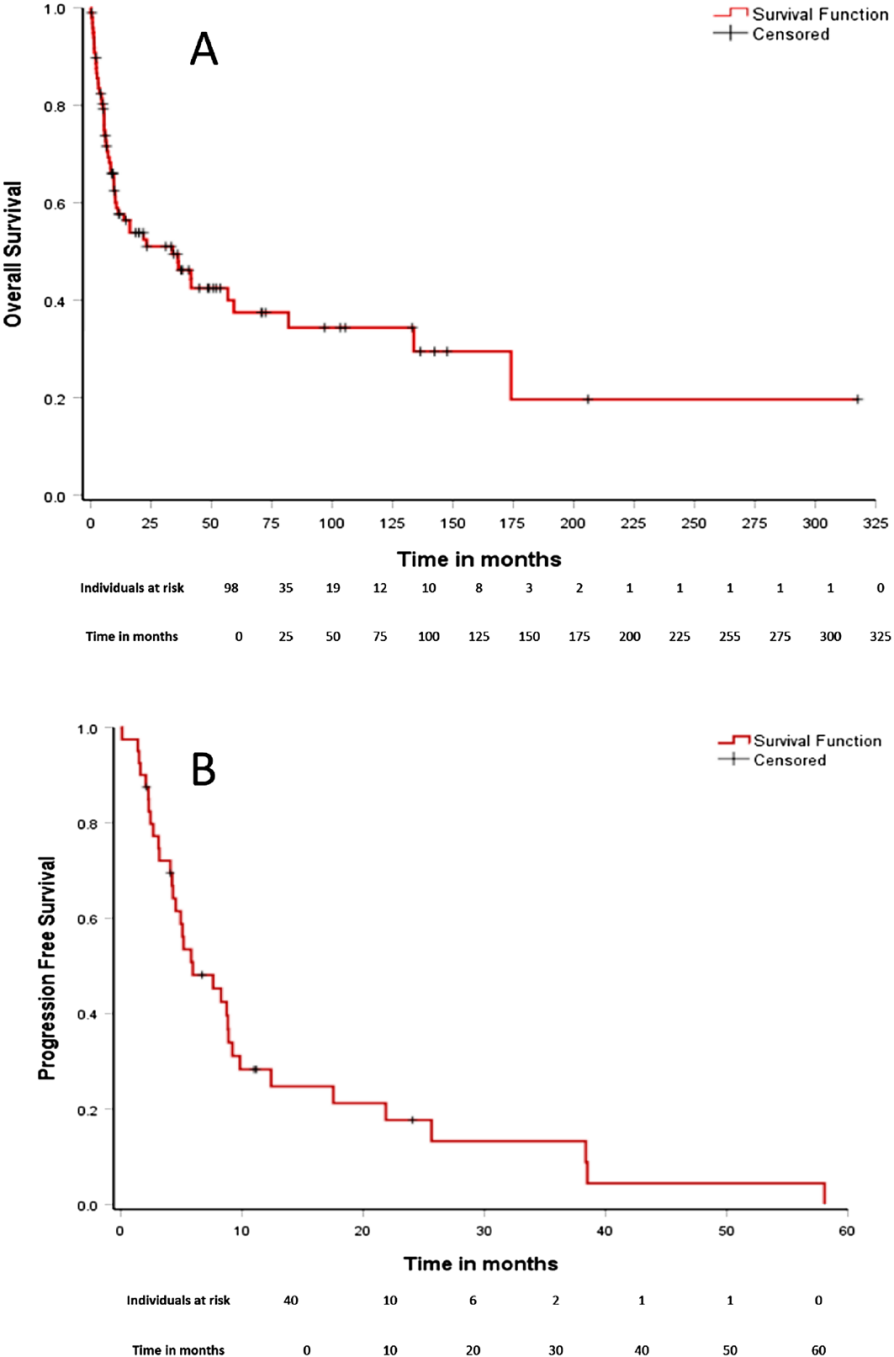
OS (**A**) e PFS (**B**) curves for 98 Brazilian patients with ENKTL.

With a median follow-up of 49.0 months (95% CI: 35.8–62.3), the median OS and PFS were 33.9 months (95% CI: 9.5–58.3) and 5.9 months (95% CI: 2.2–9.6), respectively. The estimated OS was 51.1% (95% CI: 40.4–61.6) at 2 years and 37.5% (95% CI: 25.7–49.3) at 5 years. The estimated PFS at 2 and 5 years were 17.7% (95% CI: 4.5–30.8) and 0%, respectively (Fig. 1).

### Outcomes according to up-front therapy

**Figure 2:**
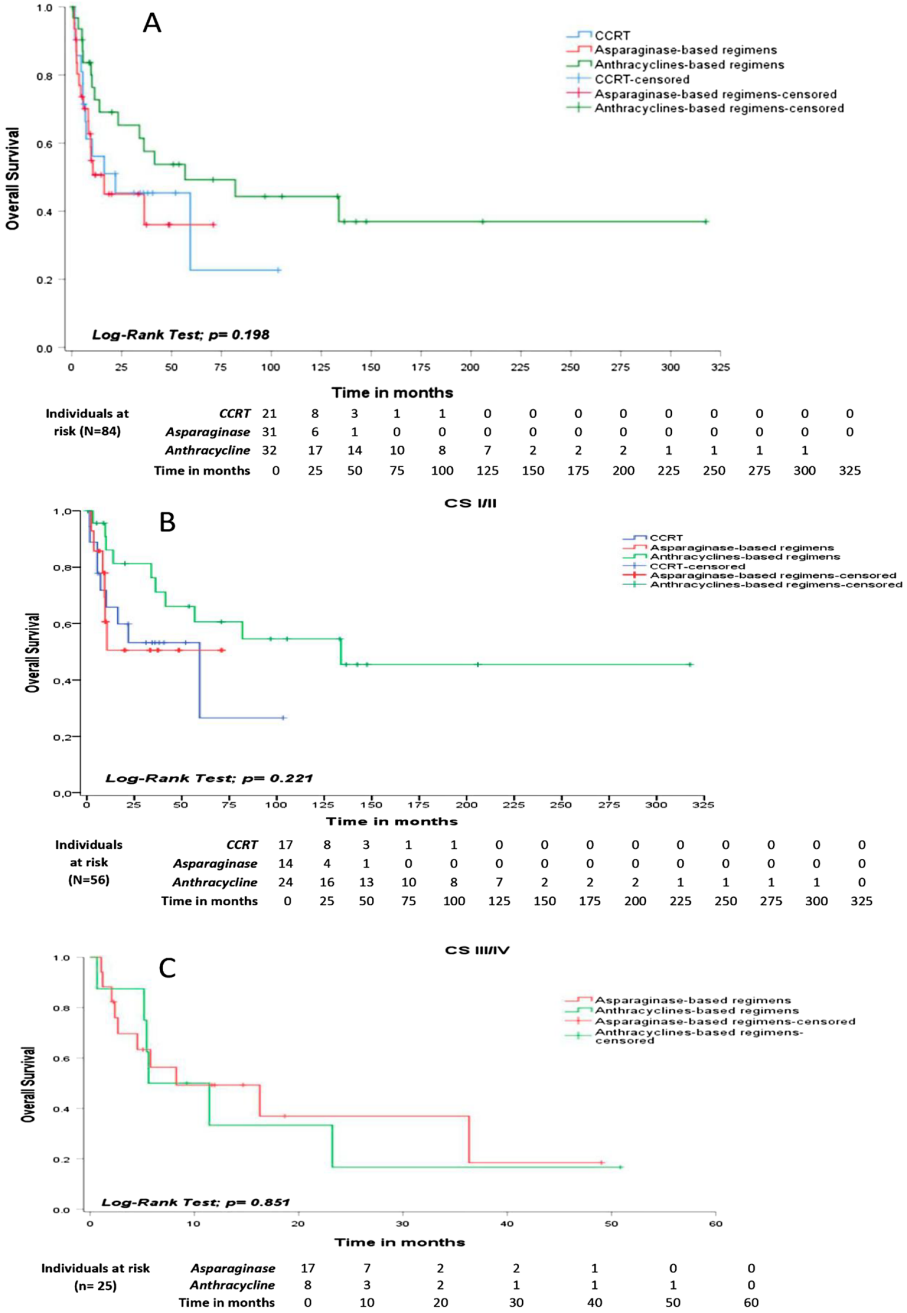
(**A**) OS according to groups of treatment: blue-line [CCRT-VIPD], green-line [SCRT based on athracyclines] and red-line [asparaginase-based regimens], *p *= 0.198. (**B**) Localized-disease (CS IE and IIE) – OS comparing CCRT/VIPD (blue-line), SCRT followed by anthracycline-based regimens (green-line) and SCRT followed by asparaginase-based chemotherapy, *p *= 0.221. (**C**) Advanced-stagedisease (CS III and IV) – OS comparing anthracycline-based regimens (green-line) and asparaginase-based regimens (red-lne), *p *= 0.851.

The ORR among the four main treatment strategies was not significantly different, *p *= 0.303 (Table 3). However, the median OS was 56.7 months (95% CI: 0.0–122.6) for EF-RT followed by CHOP-like (SCRT modality), 21.8 months (95% CI: 0.0–54.2) for CCRT-VIPD and 16.2 months (95% CI: 0.0–39.5) for asparaginase-based protocols, *p *= 0.198. The estimated OS at 2 years was 65.2% (95% CI: 47.5–82.8) for SCRT based on anthracyclines, 45.4% (95% CI: 23.4–67.3) for CCRT-VIPD and 45.0% (95% CI: 25.4–64.6) for asparaginase-based chemotherapy (Fig. 2A). There was no statistically significant difference for PFS among these different groups, *p *= 0.418.

**Table 3:** Up-front therapy modalities, responses e toxicities for 89 ENKTCL Brazilian patients.

Variable	CCRT/VIPD	CHOP-like	Asparaginase	Others	*p-*value
	N = 21, 23.6%	N = 32, 36.0%	N = 31, 34.8%	N = 5, 5.6%	
Overall response rate	09 (42.9%)	19 (59.4%)	14 (45.2%)	02 (40.0%)	0.303
Overall mortality	12 (57.1%)	16 (50.0%)	16 (51.6%)	03 (60.0%)	0.944
Induction mortality	10 (47.6%)	04 (12.5%)	07 (22.5%)	01 (20.0%)	0.034
Thrombocytopenia*	11 (52.4%)	07 (21.9%)	08 (25.8%)	02 (40.0%)	0.100
Neutropenia*	16 (76.2%)	15 (46.9%)	13 (41.9%)	03 (60.0%)	0.075
Febrile neutropenia	13 (61.9%)	08 (25.0%)	11 (35.5%)	01 (20.0%)	0.043
Mucositis*	14 (66.7%)	02 (6.30%)	10 (32.3%)	01 (20.0%)	< 0.001

*The toxicities displayed were considered for grades 3 or 4 according to CTCAE version 4.0.

**Figure 3:**
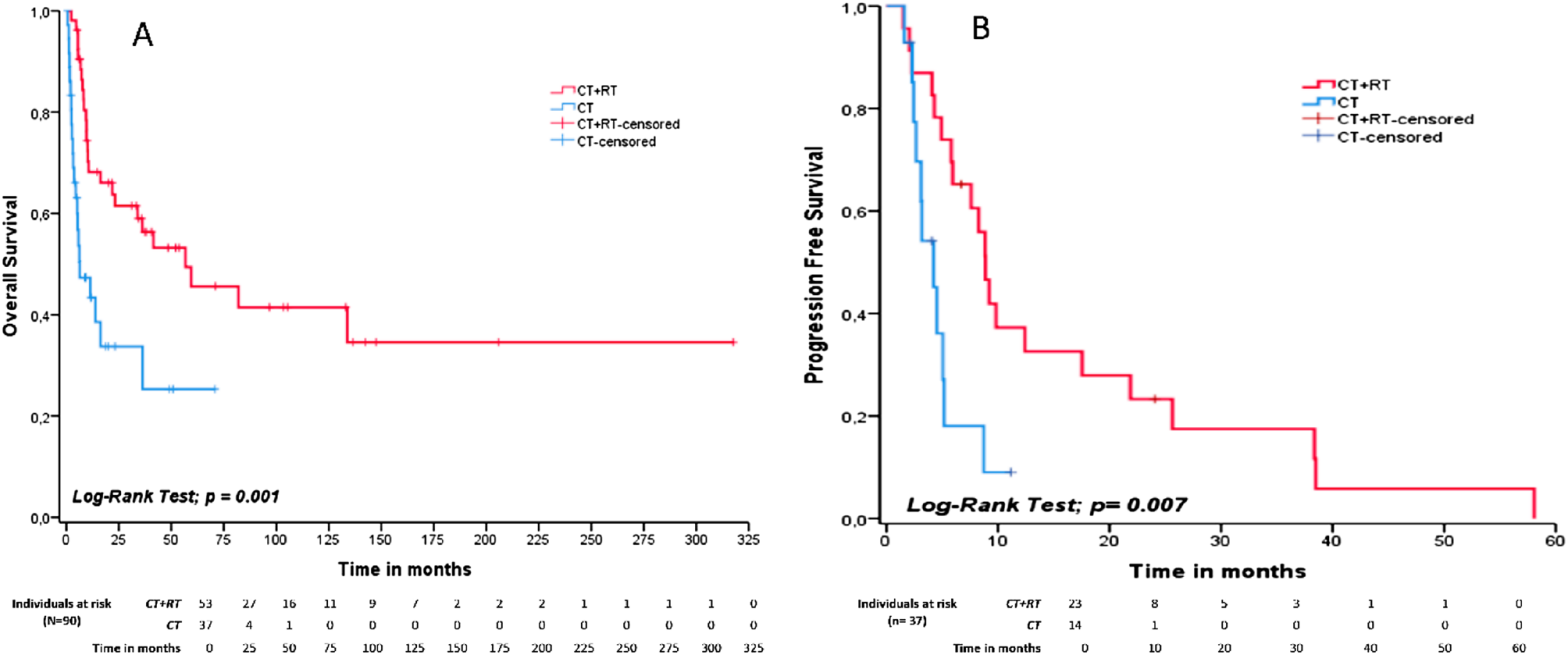
OS (**A**) and PFS (**B**) for 90 Brazilian patients with ENKTL comparing up-front treatment modalities. Red-line represents patients treated with CRT and blue-line represents cases treated with chemotherapy alone.

The median OS was 56.8 months (95% CI: 9.2–104.3) for patients treated with CRT and only 6.4 months (95% CI: 0.0–13.9) for those receiving chemotherapy alone, *p *= 0.001. The estimated 2-year OS was 61.5% (95% CI: 47.7–75.2) for CRT and 33.7% (95% CI: 15.8–51.5) for chemotherapy alone. Similarly, the median PFS was 8.8 months (95% CI: 7.5–10.2) for CRT and 4.2 months (95% CI: 2.7–5.8) for isolated chemotherapy, *p *= 0.007. The estimated 2-year PFS was 23.3% (95% CI: 5.5–41.1%) and 0% for CRT and for isolated chemotherapy, respectively (Fig. 3).

Among early-stage disease (IE/IIE) patients submitted to treatment, the median OS was 59.3 months (95% CI: 9.1–109.6) for patients treated with CCRT-VIPD (N = 18), 39.6 months (95% CI: 21.3–57.9) for patients treated with EF-RT plus asparaginase-based regimens (N = 14) and 133.7 months (95% CI: 108.1–235.7) for EF-RT followed by CHOP-like regimens (N = 24), *p *= 0.221. The estimated 2-year OS was 53.2% (95% CI: 29.3–77.1) for CCRT-VIPD, 50.5% (95% CI: 21.4–79.5) for EF-RT plus asparaginase-based chemo and 81.3% (95% CI: 64.8–97.7) for SCRT based on CHOP-like regimens (Fig. 2B). There was no statistically significant difference for PFS between these three treatment modalities among early-stage ENKTL patients, *p *= 0.671.

Among advanced-stage disease patients (III/IV) submitted to up-front therapy, the median OS was 8.2 months (95% CI: 0.0–21.8) for asparaginase-based regimens (N = 17) and 3.6 months (95% CI: 0.0–12.7) for those treated with anthracyclines (N = 8), *p *= 0.851 (Fig. 2C). The estimated OS at 2 years was 37.0% (95% CI: 8.9–65.0) for asparaginase-based chemotherapy and 33.3% (95% CI: 1.9–68.5) for CHOP-like regimens. There was no significant difference for PFS between these groups, *p *= 0.494.

### Safety and toxicity

The overall mortality rate was 55.1% [54/98] (95% CI: 45.2-64.7). The majority of deaths (22/54 – 40.7%) occurred due to disease progression, 18/54 (33.0%) were due to infectious complications and 14/54 (25.9%) were secondary to cardiovascular disease or other neoplasms (Table 3). The early-death rate (< 100 days from the diagnosis) was 29.6% (95% CI: 18.6–42.6). There was no statistically significant difference in overall mortality among the different treatment arms, *p *= 0.944.

Of note, early-deaths were more frequent in the CCRT-VIPD group (*p *= 0.034), as well as grade 3/4 neutropenia (76.2%) than with CHOP-like regimens (46.9%), asparaginase-based regimens (41.9%) or others anti-MDR drug protocols (60.0%), *p *= 0.075. Similarly, febrile neutropenia was higher with CCRT-VIPD (61.9%) in comparison to CHOP-like regimens (25.0%), asparaginase-based (35.5%) or others anti-MDR agent regimens (20.0%), *p *= 0.043. No differences were observed for grade 3/4 thrombocytopenia with CCRT-VIPD (52.4%) in comparison to 21.9% with CHOP-like, 25.8% with asparaginase and 40.0% with other anti-MDR agent’s regimens, *p *= 0.100. Grade 3/4 mucositis was more frequent in patients treated with CCRT-VIPD (66.7%) than CHOP-like regimens (6.3%), asparaginase-based regimens (32.3%) and others anti-MDR drug regimens (20.0%), *p *< 0.001 (Table 3).

### Prognostic factors – univariate and multivariate analysis

Table 4 presents univariate and multivariate analysis for OS, PFS and POD-24. In univariate analysis, advanced-stage disease III/IV (HR: 3.170, 95% CI:1.791–5.609, *p *< 0.001), EBV viral load detected in peripheral blood by qRT-PCR (HR: 1.970, 95% CI:1.033–3.757, *p *= 0.039), Hb < 120 g/L (HR: 2.484, 95% CI: 1.380–4.472, *p *= 0.002), albumin < 3.5 g/dL (HR: 2.903, 95% CI:1.480–5.692, *p *= 0.002), high-risk PINK score (HR: 3.176, 95% CI:1.768–5.703, *p *< 0.001), B-symptoms (HR: 1.803, 95% CI:1.097–3.320, *p *= 0.048), relapsed or refractory (R/R) disease (HR: 3.100, 95% CI:1.753–5.185, *p *< 0.001), radiotherapy omission (HR: 2.875, 95% CI:1.586–5.214, *p *= 0.001), ECOG ≥ 2 (HR:2.116, 95% CI: 1.172–3.820, *p *= 0.013), lymphopenia (HR: 2.080, 95% CI: 1.171–3.697, *p *= 0.013) and POD-24 (HR: 3.782, 95% CI: 2.141–7.004, *p *= 0.044) were predictors for decreased OS.

**Table 4:** Prognostic factors for OS, PFS and POD-24 – univariate and multivariate analysis.

	Univariate analysis					Multivariate analysis			
	OS	PFS	POD-24	OS		PFS		POD 24	
	*P-*value			Hazard ratio (95%CI)	*P-*value	Hazard ratio (95%CI)	*P-*value	Hazard ratio (95%CI)	*P-*value
III/IV Stage	< 0.001	0.034	0.007					3.10 (1.3–7.0)	0.007
EBV viral load	0.039	–	–						
Hb < 120 g/L	0.002	–	–	2.05 (1.0–4.0)	0.03				
Albumin < 3.5 g/dL	0.002	–	–						
High-risk PINK score	< 0.001	0.011	0.005						
B symptoms	0.048	–	–						
R/R disease	< .001	–	–	2.98 (1.5–5.6)	0.001				
No radiotherapy	0.001	0.006	0.019	3.15 (1.6–6.0)	0.001	2.79 (1.0–7.1)	0.023		
ECOG ≥ 2	0.013	–	–						
Lymphopenia	0.013	0.005	0.038			3.60 (1.3–9.6)	0.012		
Bulky disease	–	–	0.044						

*R/R disease* relapsed/refractory disease, *ECOG* Eastern Cooperative Oncology Group – Performance status scale.

Age (*p *= 0.336), gender (*p *= 0.656), extra-nasal disease (*p *= 0.969), ≥ 2 comorbidities (*p *= 0.829), high β2-MG (*p *= 0.458), high LDH (*p *= 0.357), leukocytosis (*p *= 0.226), neutropenia (*p *= 0.619), monocytosis (*p *= 0.419), hypogammaglobulinemia (*p *= 0.353), high-risk PINK-E index (*p *= 0.864), tumor site infection (*p *= 0.925), tumor necrosis ≥ 50% (*p *= 0.834), CD30 expression (*p *= 0.728) and bulky disease (*p *= 0.080) were not predictive of OS.

Advanced-stage disease (III/IV) (HR: 2.147, 95% CI: 1.064–4.748, *p *= 0.034), high-risk PINK score (HR: 2.833, 95% CI: 1.266–6.339, *p *= 0.011), lymphopenia (HR: 3.633, 95% CI: 1.473–8.960, *p *= 0.005) and radiotherapy omission (HR: 3.186, 95% CI: 1.385–7.333, *p *= 0.006) were associated with decreased PFS. Likewise, high-risk PINK score (HR: 2.743, 95% CI: 1.349–5.578, *p *= 0.005), bulky disease (HR: 2.672, 95% CI: 1.026–6.957, *p *= 0.044), advanced-stage III/IV (HR: 2.545, 95% CI: 1.290–5.019, *p *= 0.007), radiotherapy omission (HR: 2.313, 95% CI: 1.146–4.668, *p *= 0.019) and lymphopenia (HR: 1.871, 955 CI: 0.921–3.804, *p *= 0.038) were predictors associated with POD-24.

The multivariate analysis showed that the independent prognostic factors for overall survival among all ENKTL patients were Hb < 120 g/L (HR: 2.052, 95% CI: 1.042–4.041, *p *= 0.038), relapsed/refractory disease (HR: 2.983, 95% CI: 1.568–5.673, *p *= 0.001) and no radiation therapy in up-front treatment (HR: 3.155, 95% CI: 1.649–6.039, *p *= 0.001). Lymphopenia (HR: 3.60, 95% CI: 1.318–9.614, *p *= 0.012) and radiotherapy omission (HR: 2.791, 95% CI: 1.088–7.161, *p *= 0.023) were independent prognostic factors for decreased PFS. Advanced-stage III/IV (HR: 3.101, 95% CI: 1.372–7.013, *p *= 0.007) was an independent predictor associated with POD-24.

## Discussion

In this first multicentric retrospective study of ENKTL in Brazil, we described clinical characteristics and outcomes of 98 patients and as reported in literature, our findings showed dismal survival, but similar to those described for patients receiving anthracycline-based regimens (30–50%)^4,33^. At a median follow-up of 49.0 months, the estimated overall survival at 2 years was 51.1% and 37.5% at 5 years.

Currently, anthracycline-based therapy, such as CHOP-like regimens are no more an optimal treatment for ENKTL^34–36^ because of the high expression of *ABCB1* (*MDR-1*) gene on ENKTL patient’s neoplastic cells^15,16^. The P-glycoprotein (Pgp) encoded by its gene acts as an efflux-pump for anthracyclines and vinca alkaloids causing resistance to these drugs^36–38^. However, as our study encompassed patients treated in a long period, a reasonable number of patients received CHOP-like regimens.

Not surprisingly, up-front EF-RT was associated with increased OS and PFS in multivariate analysis of our cohort. The OS at 2 years was 61.5% for patients receiving chemoradiotherapy and only 33.7% for those treated with chemotherapy alone, *p *= 0.001. Similarly, estimated 2-year PFS was 23.3% and 0% for cases treated with chemoradiotherapy and isolated chemotherapy, respectively, *p *= 0.007. In agreement with our data, previous studies demonstrated better overall survival for early-stage ENKTL treated with high-dose EF radiotherapy in comparison to chemotherapy alone^17,39^. Furthermore, chemoradiation with non-anthracycline regimens has also been associated with higher overall response rate and overall survival^17,22,23,40^. Even radiotherapy alone promotes long time control in limited-stage and low-risk ENKTL^41–43^.

Unexpectedly, patients submitted to concurrent chemotherapy plus radiotherapy approach (CCRT-VIPD) presented worse outcomes in comparison to those previously reported. In the Brazilian cohort, 21/89 (23.6%) early-stage ENKTL patients received EF-radiotherapy concurrent with cisplatin once a week followed by three cycles of VIPD (etoposide, ifosfamide, cisplatin and dexamethasone) regimen (CCRT-VIPD), as proposed by the *Korean Consortium for Improving Survival of Lymphoma* in a phase II study^25^**.** In this study, the authors reported 83.3% of ORR, 80% of CR and 3-year OS and PFS of 86.2% and 85.1%, respectively. However, in our cohort the median OS was 59.3 months for early-stage ENKTL treated with CCRT-VIPD and 133.7 months for sequential chemoradiotherapy with CHOP-like regimens, *p *= 0.113. The 2-year OS was 53.2% for CCRT-VIPD and 81.3% for EF-RT followed by anthracycline-based chemotherapy. Kim et al. (2009) reported that 12/29 (41%) patients had grade 4 neutropenia and two treatment-related deaths in patients using CCRT-VIPD^25^.

After these results, the same group developed a less aggressive version of this protocol (CCRT-VIDL) for early-stage ENKTL^44^. We also observed high-rates of adverse events related to treatment in our patients receiving CCRT-VIPD. In fact, 12/21 (57.1%) patients submitted to this approach died, mainly during the induction phase (7/21 – 46.6%). The early-mortality rate in patients submitted to CCRT-VIPD was significantly higher when compared to the other therapeutic modalities, *p *= 0.070, as summarized in Table 3. Neutropenia (*p *= 0.075), febrile neutropenia (*p *= 0.043), and mucositis (*p *< 0.001) were also more frequent in this group, corroborating the high early mortality observed. In opposition to Kim et al. (2009), our patients were treated in a real-world setting without any selection and one quarter of our cohort presented poor performance status by ECOG, 20.4% were older than 60 years and 41.8% presented tumor infection at diagnosis. Furthermore, Kim et al. (2009) evaluated only 30 selected patients, being 86.7% under 60 years and 100% of low risk^25^.

Other concurrent chemoradiotherapy regimens, such as RT/DeVIC, associating dexamethasone, etoposide, ifosfamide and carboplatin, ESHAP (etoposide, methylprednisolone, high-dose ara-c and cisplatin) and DEP (dexamethasone, etoposide, and cisplatin) have also been explored with favorable outcomes for ENKTL. However, they also resulted in excessive hematologic and non-hematologic toxicities^45–47^.

More recently, aiming to reduce the toxicity of the CCRT regimens, other sequential regimens (SCRT) with chemotherapy followed by radiotherapy have been developed for early-stage ENKTL^48,49^. These approaches provide high rates of response, long-term disease control and low toxicity even for elderly and frail patients^39,50^. Wang et al. conducted a phase II study targeting early-stage ENKTL, alternating two cycles of gemcitabine, l-asparaginase and oxaliplatin (GELOX) with local radiotherapy at a dose of 56 Gy followed by two or four additional cycles of GELOX. The authors showed an ORR of 96% and 74% of CR^20^. Long-term follow-up showed a 5-year OS of 85% and PFS of 74%^20^. Similar regimens replacing L-asparaginase by peg-asparaginase (P-GEMOX) were also associated with low toxicity^27,51,52^.

Due to its high radiosensitivity, RT is currently considered the cornerstone of limited-stage ENKTL therapy. Although some patients really can be cured with isolated high-dose EF-RT (50–60 Gy), this therapeutic modality is usually reserved for elderly patients, for those with poor performance status, or with severe comorbidities. In fact, different groups have shown that the combination of high-dose EF-RT plus multidrug chemotherapy increases response and survival rates in early-stage ENKTL patients compared to radiotherapy alone^27,43,51,53^**.**

Previous studies indicated that RT with doses lower than 50 Gy were associated with inferior response and outcomes^54^. However, recent trials demonstrate that lower doses of RT (< 50 Gy) do not compromise patient outcomes with early-stage ENKTL, particularly when associated with chemotherapy regimens based on asparaginase, same as for patients in complete remission after chemotherapy^55^. Such approach aims to reduce the toxicity associated with high-dose EF-RT.

In our study, the median dose of radiotherapy was 54 Gy (IqR 25–75%: 50–60 Gy). Some patients underwent EF-RT with lower doses such as 40–45 Gy. However, in our cohort, only 15% (14/93) of cases had early stage ENKTL and received SCRT based on asparaginase. In addition, we had a high percentage of patients receiving anthracyclines. The higher median RT dose employed in our casuistic can be partially explained by the inclusion of patients followed during almost two-decades, with many being treated in an era where doses greater than 50 Gy were believed optimal for early-stage ENKTL.

Although CRT based on anti-MDR agents has become the current standard of care for managing early-stage ENKTL patients, our real-life study was unable to demonstrate benefit of this therapeutic modality over SCRT based on anthracyclines. Brazilian patients with early-stage (IE/IIE) ENKTL had an estimated 2-year OS of 81.3% when treated with SCRT followed by CHOP-like regimens and only 50.5% for those submitted to CRT based on asparaginase, *p *= 0.221. Such therapeutic recommendations were based on retrospective, phase II trials, involving low number of selected patients. Therefore, we believe that the characteristics of our cohort, involving patients in a real-life setting, with unfavorable clinical-biological findings and compromised socioeconomic conditions, have contributed to the lack of clinical benefit of asparaginase-based CRT regimens observed in our early-stage ENKTL patients.

In our casuistic, the estimated 2-year OS was 37.0% for advanced-disease using asparaginase-based protocols and 33.0% for anthracycline-based regimens, *p *= 0.851. These findings differ from literature, where previous studies demonstrated longer survival for extra-nasal and advanced-stage ENKTL receiving asparaginase-based schemes as opposed to non-asparaginase regimens. Nevertheless, OS of advanced-stage ENKTL remains poor, even when treated with asparaginase-based regimens^39,50^. Again, we hypothesize that these results are supported by our unselected cohort, composed by patients treated in a real-world setting, and presenting high toxicity to regimens including anti-MDR agents.

In our study, the most common regimen employed for advanced-stage disease and extranasal ENKTL was SMILE (methylprednisolone, methotrexate, ifosfamide, l-asparaginase and etoposide). In fact, it was the first regimen developed in cooperative clinical trials in East Asia to explore a more effective induction therapy before ASCT^26^. This was the first chemotherapeutic regimen that has demonstrated efficacy in clinical trials in newly diagnosed stage IV ENKTL^26^. More recently, Wang X et al. (2021), conducted other phase II study, where 42 advanced-stage ENKTL patients were prospectively treated with DDGP (dexamethasone, cisplatin, gemcitabine and peg-asparaginase) or SMILE. The authors reported higher CR in the DDGP (71%) arm than in the SMILE (29%), *p *= 0.005. The 1-year PFS was also higher for DDGP (86%) than for SMILE (38%), *p *= 0.006. The DDGP regimen showed better tolerability, less leukopenia (*p *= 0.030) and less allergic reactions (*p *= 0.015)^21^. In opposition, SMILE was associated with higher rate of grade 3/4 adverse events, including diarrhea, mucositis, heart failure, arrhythmias and deaths related to treatment^21^.

Different from previous studies, we demonstrated that anemia and lymphopenia were independently associated with shorter OS and PFS, respectively, in multivariate analysis. However, in concordance with other authors, advanced stage III/IV remained an independent predictive factor for adverse outcomes^53,56–59^. As expected, PINK score was not predictive of prognosis in multivariate analysis in our cohort. In fact, this score was identified as predictive of prognosis for patients that underwent non-anthracycline-based regimens^60^.

As our group was interested in predictive factors for early relapse (i.e., with relapse within 24 months fom the beginning of therapy), we included this endpoint in our analysis. Fortunately, we were able to show that advanced-stage disease was associated with POD-24 in our cohort of ENKTL. Yamaguchi M et al. (2018), investigated POD-24 in 165 patients and they did not find any variable predictive of POD-24, but they concluded that POD-24 is a strong endpoint indicator of survival in ENKTL^33^.

Similar to literature, hemophagocytic syndrome at diagnosis was infrequent in our casuistic ^53,61^. Nonetheless, our cohort showed unexpectedly high leukopenia frequency (23.4%) and thrombocytopenia frequency (34.6%), probably not related to bone marrow infiltration or splenomegaly, since both were rare in our patients. Hence, we interrogated if these cytopenias may be actually hemophagocytosis related. However, we were unable to demonstrate this, given the retrospective nature of our cohort and missing data for variables such as triglycerides, ferritin, and fibrinogen levels. However, we assume that hemophagocytic syndrome diagnosis was underestimated in our study. In fact, almost half of the lymphoma-associated HLH cases have been reported to be correlated with EBV-associated lymphoid malignancies, as ENKTL^62^.

## Conclusion

In this first Brazilian multicenter report of ENKTL, we found that our patients’s characteristics were similar to other casuistics previously reported. High-dose EF-RT proved to be the cornerstone for the treatment of early-stage disease, and its omission was associated with decreased OS and PFS. In our real-life study, CCRT regimens were poorly tolerated, and protocols based on anti-MDR agents were not associated with better outcomes neither in early nor in advanced-stage diseases. We also found that anemia, lymphopenia, and advanced stage III/IV were independent predictors associated with poor clinical outcomes.

## Data Availability

All data generated and analyzed during this study were included in this published article. The raw data for this study are in possession of the author and may be fully available in the event of a request to the author via e-mail.
